# Dapagliflozin reduces systemic inflammation in patients with type 2 diabetes without known heart failure

**DOI:** 10.1186/s12933-024-02294-z

**Published:** 2024-06-07

**Authors:** Dennis D. Wang, Anna V. Naumova, Daniel Isquith, Jamie Sapp, Kim A. Huynh, Isabella Tucker, Niranjan Balu, Anna Voronyuk, Baocheng Chu, Karen Ordovas, Charles Maynard, Rong Tian, Xue-Qiao Zhao, Francis Kim

**Affiliations:** 1 Division of Cardiology, Department of Medicine, University of Washington, Seattle, WA USA; 2 Department of Radiology, University of Washington, Seattle, WA USA; 3 Department of Anesthesiology and Pain Medicine, University of Washington, Seattle, WA USA; 4 Department of Health Systems and Population Health, University of Washington, Seattle, WA USA; 5 University of Washington, 850 Republican St, Box 358055, Seattle, WA 98104 USA

**Keywords:** Type 2 diabetes, Inflammation, IL-1B, PBMC respiration, CMRI, Cardiac fibrosis, SGLT2 inhibitor

## Abstract

**Objective:**

Sodium glucose cotransporter 2 (SGLT2) inhibitors significantly improve cardiovascular outcomes in diabetic patients; however, the mechanism is unclear. We hypothesized that dapagliflozin improves cardiac outcomes via beneficial effects on systemic and cardiac inflammation and cardiac fibrosis.

**Research and design methods:**

This randomized placebo-controlled clinical trial enrolled 62 adult patients (mean age 62, 17% female) with type 2 diabetes (T2D) without known heart failure. Subjects were randomized to 12 months of daily 10 mg dapagliflozin or placebo. For all patients, blood/plasma samples and cardiac magnetic resonance imaging (CMRI) were obtained at time of randomization and at the end of 12 months. Systemic inflammation was assessed by plasma IL-1B, TNFα, IL-6 and ketone levels and PBMC mitochondrial respiration, an emerging marker of sterile inflammation. Global myocardial strain was assessed by feature tracking; cardiac fibrosis was assessed by T1 mapping to calculate extracellular volume fraction (ECV); and cardiac tissue inflammation was assessed by T2 mapping.

**Results:**

Between the baseline and 12-month time point, plasma IL-1B was reduced (− 1.8 pg/mL, *P* = 0.003) while ketones were increased (0.26 mM, *P* = 0.0001) in patients randomized to dapagliflozin. PBMC maximal oxygen consumption rate (OCR) decreased over the 12-month period in the placebo group but did not change in patients receiving dapagliflozin (− 158.9 pmole/min/10^6^ cells, *P* = 0.0497 vs. − 5.2 pmole/min/10^6^ cells, *P* = 0.41), a finding consistent with an anti-inflammatory effect of SGLT2i. Global myocardial strain, ECV and T2 relaxation time did not change in both study groups.

**Clinical Trial.gov Registration:**

NCT03782259.

**Supplementary Information:**

The online version contains supplementary material available at 10.1186/s12933-024-02294-z.

## Introduction

Sodium glucose cotransporter 2 (SGLT2) inhibitors prevent glucose reabsorption in the kidneys, increasing urinary glucose excretion and lowering plasma glucose. In the EMPA-REG OUTCOME and CANVAS trials, SGLT2 inhibitors have been shown to significantly reduce cardiovascular events in T2D patients [1, 2]. These benefits are independent of glycemic control. While the mechanism by which SGLT2 inhibition improves cardiovascular outcomes in T2D remains elusive, there is ample evidence in pre-clinical and clinical studies to suggest that SGLT2 inhibition is associated with a reduction in inflammation [3, 4]. Recently, subgroup analysis of the CANTOS trial suggests that anti- interleukin (IL)-1B therapy may reduce heart failure (HF) hospitalizations in myocardial infarction (MI) patients with elevated high sensitivity C-reactive protein (CRP) [5], implicating IL-1B as a key mediator in cardiac inflammation. Experimental T2D animal models suggest that SGLT2 inhibitors increase the rate of glucose and fatty acid oxidation leading to an increase in circulating ketone levels, which was shown to inhibit NLRP3 inflammasome activation, resulting in reduced IL-1B production in macrophages [6]. Taken together, it is hypothesized that SGLT2 inhibitor’s effect in improving cardiac outcomes is mediated through antagonizing IL-1B. However, to date, there has been no double-blind placebo controlled randomized trials to demonstrate that SGLT2 inhibition reduces systemic IL-1B in T2D patients.

Cardiac fibrosis, which is known to adversely affect diastolic function [7, 8], plays an important role in the pathogenesis of diabetic cardiomyopathy [9]. Cardiac magnetic resonance imaging (CMRI) using T1-mapping is capable of quantifying myocardial extracellular volume (ECV), a surrogate of fibrosis, with excellent inter- and intra-observer variability and could, therefore, be potentially employed for investigations in diabetic cardiomyopathy [10]. A recent study [11] showed that ECV by T1-mapping increased as the duration of diabetes increased from 3 to 9 months in diabetic rabbits, consistent with the changes in myocardial fibrosis verified by pathology. In addition, CMRI’s feature tracking technique assesses regional functional abnormalities, and T2-mapping evaluates myocardial edema from injury and inflammation [12].

The aims of this double-blind placebo-controlled study are to investigate whether dapagliflozin treatment for 12 months could reduce systemic and myocardial inflammation and improve myocardial fibrosis in T2D patients.

## Research design and methods

### Study setting and patients

This double-blind, randomized trial assigned adults with T2D to placebo or 10 mg dapagliflozin daily for 12 months. The study began on 2/26/2019; initial screening of patients and follow up visits occurred at the Clinical Atherosclerosis Research Laboratory at Harborview Medical Center, University of Washington. The study was completed on 11/16/2022.

### Inclusion criteria

Age > 18; T2D history > 5 years; Hemoglobin A1c (7–10%), glucose control medication: insulin, metformin, and/or sulfonylurea.

### Exclusion criteria

Current use of SGLT2 inhibitor; hypersensitivity to SGLT2 inhibitor; diagnosis of heart failure; contraindications to MRI; eGFR < 45 ml/min/1.73m^2^; unstable or progressing renal disease; SBP < 100 mmHg; severe hepatic disease (Child-Pugh Class C); active hepatitis B or C, CV disease within 3 months before enrollment (myocardial infarction; CABG, coronary intervention; TIA; stroke, PAD); Bladder cancer; or high risk of diabetic ketoacidosis, high risk of fracture (osteoporosis, osteopenia).

### Enrollment and randomization

**Fig. 1:**
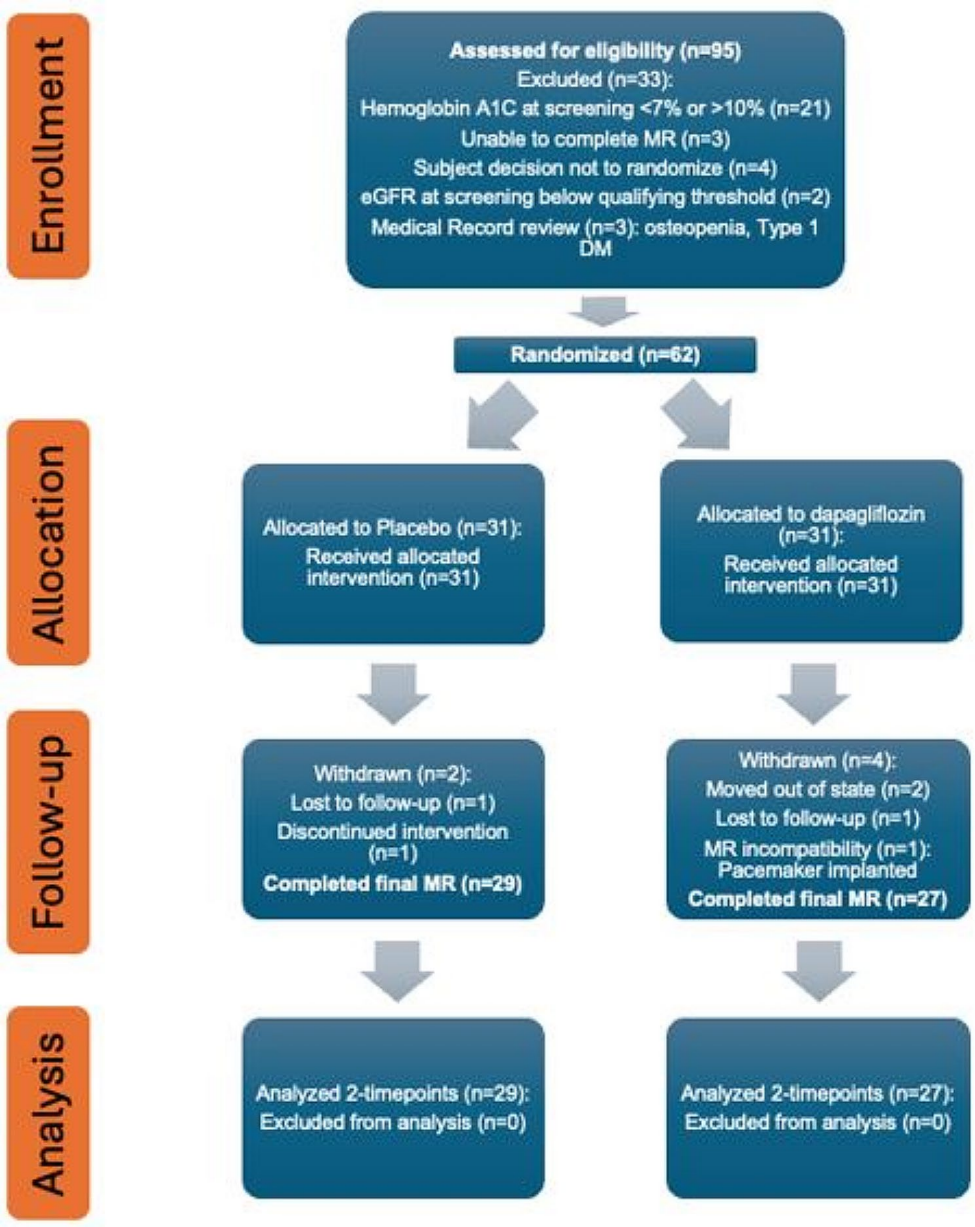
Trial CONSORT Flow Diagram. MR, magnetic resonance; DM, diabetes mellites

T2D patients without known heart failure diagnosis (by ICD code) were enrolled in the study. Transthoracic echocardiogram (TTE) was not part of the screening protocol and only a small percentage of the study subjects underwent TTE as per standard of care prior to enrollment. During the enrollment period, 95 patients were assessed for eligibility, 62 patients were randomized, 56 patients completed the study protocol (Fig. 1). Randomization was stratified according to use of glucagon-like peptide (GLP-1) and angiotensin-II receptor blockers (ARBs).

### Study intervention

Following screening visit and informed consent, patients were randomized 1:1 to placebo or 10 mg dapagliflozin daily for 12 months. Randomization was performed by the Investigational Drug Services at Harborview Medical Center. During the randomization visit, blood samples were collected for peripheral blood mononuclear cell (PBMC) respiration assessment and plasma samples were collected for cytokine and ketone measurements. Patients also received a baseline CMRI and laboratory evaluation. Patients had clinical visits at 3, 6, 9 months, and at the 12-month visit patients underwent a final CMRI along with blood and plasma sample collection.

### Outcomes

Primary outcomes: Changes in global myocardial strain and ECV as assessed by T1 mapping (baseline to 12 months).

Secondary outcomes: Changes in plasma IL-1B, TNFα, IL-6, IL-10, plasma ketones, T2 relaxation time (baseline to 12 months).

Exploratory outcome: PBMC mitochondrial basal and maximal oxygen consumption rate determined by Seahorse XF Analyzer.

### Plasma cytokine and Ketone Quantifications

Plasma samples were obtained from whole blood collected in EDTA-containing vacutainers post 2000 g x 10’ at 4 °C and stored in − 80 °C. Plasma concentrations of cytokines were determined by ELISA following manufacturer’s protocol (Biolegend): IL-1B (Cat: 437,004), TNFα (Cat: 430,204), IL-6 (Cat: 430,504), and IL-10 (Cat: 430,601). Plasma concentrations of β-hydroxybutyrate and acetoacetate were determined by EnzyChrom™ Ketone Body Assay Kit following manufacturer’s protocol (BioAssay Systems, Cat: EKBD-100).

### PBMC Oxygen Consumption Rate (OCR) Measurement

PBMC was isolated from whole blood collected in acid-citrate-dextrose vacutainers post density gradient (Histopaque-1077, Sigma-Aldrich Cat: 10,771) centrifugation. Freshly isolated PBMCs were resuspended in Seahorse XF medium (Cat: 102353-100) and then plated (10^6^ cells per well) onto Seahorse XFe24 cell culture plate. PBMC mitochondrial respiratory function was assessed by measuring the OCR at basal and maximal stimulated conditions using Seahorse XFe24 Analyzer as described previously [13].

### Cardiac magnetic resonance imaging

CMRI examination was done at a 3T clinical whole-body scanner (Ingenia, Phillips®) located at the BioMolecular Imaging Center (BMIC) at the University of Washington, South Lake Union campus. CMRI protocol included: steady state free precession (SSFP) cine imaging to measure heart LV chamber volumes (assessing dilatation and hypertrophy), contractile function and myocardial strain; naïve and post-contrast T1 mapping and ECV fraction to assess changes in diffused myocardial fibrosis; T2 mapping to assess myocardial inflammation; T2* mapping to assess iron deposition; Late gadolinium enhancement for visualize focal fibrosis.

All imaging acquisitions were done with ECG gating and breath hold technique. Imaging parameters are shown in Supplemental Table 1.

### Image processing and analysis

Volumetric LV analysis and analysis of quantitative maps (T1, T2, T2*) were performed using Philips IntelliSpace Portal (ISP) software. Volumetric parameters are reported as indexes, after adjustment for body surface area. Variables are compared to normal age specific ranges reported in the literature.

ECV maps were generated offline using MATLAB software. ECV was calculated from native and post-contrast T1 values for blood and myocardial tissue, the partition coefficient lambda (λ), and hematocrit using the following formulas: ECV = λ(1-hematocrit); λ = (1/T1 myocardium post-contrast-1/T1 myocardium-native)/(1/T1 blood post-contrast-1/T1 blood-native).

**Fig. 2:**
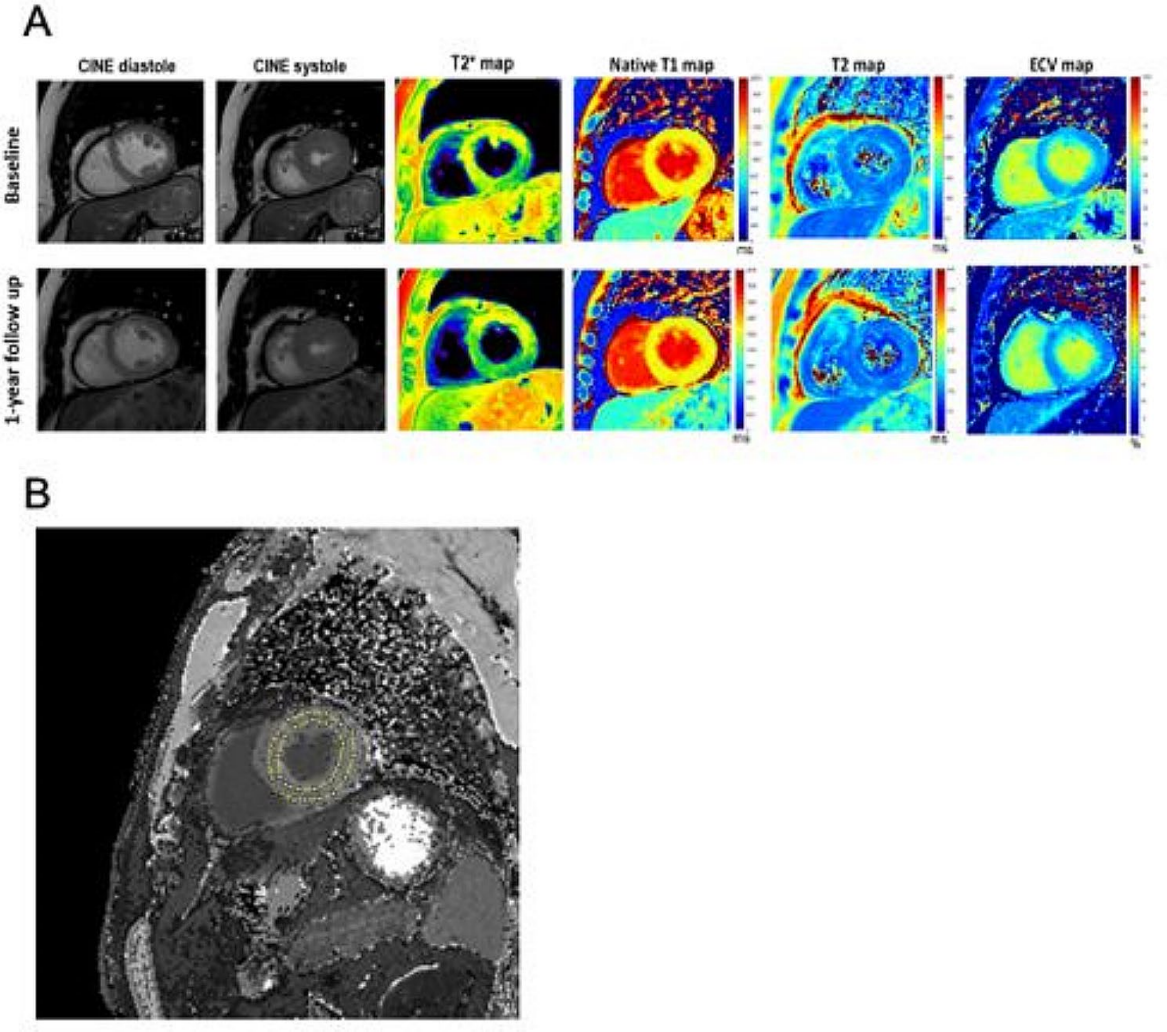
(**A**) Representative CMRI quantitative maps of T2D patient scanned at the baseline and 1-year follow up. (**B**) Example of region of interest (ROI) placement to the left ventricle of the post-contrast T_1_ map for evaluation the parametric mapping values. The C-shape ROI included the whole circumference of the left ventricle. The ROI was placed at about 1 mm away from the LV edges to exclude signal contamination from blood and surrounding tissues

For precise calculation of parametric mapping values, the ROI was placed in the mid-ventricular short-axis slice of the left ventricle covering the whole circumference of the left ventricle with 1 mm indentation from the LV edge to exclude signal contamination from blood and surrounding tissues [14]. All parametric maps had the same ROI shape and location as shown in Fig. 2B.

Feature tracking was performed using Circle Cardiovascular Software (cvi-42, Circle Cardiovascular Imaging Inc., Calgary, Alberta, Canada) to measure myocardial strain and strain rate from the bSSFP short-axis and long-axis cine images. Long-axis cine images were further used to compute global myocardial longitudinal strain. Short-axis images were used to compute circumferential and radial strain and strain rate. The global values were obtained through averaging the values according to an American Heart Association 17-segment model [15].

### Statistical analysis

For systemic inflammatory endpoints (plasma cytokines, plasma ketones, and PBMC OCR), we compared baseline and 1-year post-intervention values in the dapagliflozin and placebo groups. P-values were determined by paired two-tailed t-test. Parametric t-test was used if distribution passes normality test, otherwise non-parametric t-test (Wilcoxon) was used.

For CMRI outcomes, we compared baseline characteristics in the dapagliflozin and placebo groups and expressed age as mean and standard deviation and categorical variables as numbers and percents. The difference between baseline and 1-year results was calculated for the primary outcomes in the dapagliflozin and placebo groups. Within each group, the difference between baseline and 1 year was assessed with the paired t-test. The differences between drug and placebo groups were compared with the Wilcoxon Rank Sum Test.

To adjust for multiple comparisons, the level of statistical significance was set at 0.0125 (0.05/4) for primary outcomes, and 0.0083 (0.05/6) for secondary outcomes.

### Ethical oversight

The trial was approved by the Institutional Review Board at the University of Washington.

## Results

### Baseline characteristics

As shown in Table 1, mean age of participants was 62 years and 17% were female. Of the participants, 61% had hypertension, 60% had hyperlipidemia, and 40% had a family history of coronary artery disease. No clinically meaningful differences were noted in the baseline characteristics between the dapagliflozin and placebo groups following randomization.

**Table 1:** Baseline characteristics

Characteristic	Dapagliflozin (*n* = 31)	Placebo (*n* = 31)
Age, (mean, standard deviation)	62 (9)	62 (11)
Women	6 (19%)	5 (16%)
Hispanic	1 (3%)	4 (13%)
**Race**		
White	23 (74%)	20 (64%)
Black	3 (10%)	3 (10%)
Asian	3 (10%)	6 (19%)
Native American	2 (6%)	1 (3%)
Not provided	0 (0%)	1 (3%)
Hypertension	19 (61%)	19 (61%)
Diabetes mellitus	31 (100%)	31 (100%)
Hyperlipidemia	21 (68%)	18 (58%)
Current smoker	2(6%)	3 (10%)
Family history MI	12 (39%)	11 (36%)
Family history stroke	14 (45%)	11 (36%)
History MI	1 (3%)	2 (6%)
Angina	1 (3%)	2 (6%)
Coronary artery bypass surgery	0 (0%)	2 (6%)
Percutaneous coronary intervention	3 (10%)	1 (3%)
**Diabetes Medications**	31 (100%)	31 (100%)
Insulin	12 (39%)	11 (36%)
Metformin	26 (84%)	26 (84%)
**Cardiac Medications**	24 (77%)	26 (84%)
Calcium channel blocker	8 (26%)	7 (23%)
Beta blocker	5 (16%)	8 (26%)
ACE-I	13 (42%)	14 (45%)
ARB	9 (29%)	10 (32%)

MI, myocardial infarction

*P* > 0.05 for all treatment group comparisons

### Cardiac MR endpoints

As shown in Table 2, ECV, a measurement of cardiac fibrosis, and myocardial strain values did not differ between baseline and at 1-year follow-up in either study group. The global radial peak strain in placebo group at 1-year trended higher in comparison to baseline (31.3 ± 10.4% vs. 27.3 ± 7.1%, *P* = 0.043, respectively). However, after adjusting for multiple comparisons (corrected standard *P* = 0.0125), the P-value of 0.043 was not statistically significant.

**Table 2:** CMRI outcomes

Outcome	Drug			Placebo			Difference (1 year–baseline)		
	Baseline	1 year	P*	Baseline	1 year	P*	Drug	Placebo	P**
ECV	27.7 (2.9)	28.4 (2.0)	0.20	28.5 (2.1)	28.7 (2.3)	0.56	0.71 (2.75) (*n* = 26)	0.24 (2.16) (*n* = 28)	0.20
Radial peak strain global	31.2 (8.7)	33.9 (9.5)	0.14	27.3 (7.1)	31.3 (10.4)	0.043	2.71 (9.38) (*n* = 27)	4.01 (10.19) (*n* = 29)	0.80
Circumferential peak strain global	− 17.8 (2.9)	− 17.7 (3.6)	0.93	− 15.7 (2.8)	− 16.7 (2.9)	0.12	0.05 (3.21) (*n* = 27)	− 0.97 (3.28) (*n* = 29)	0.43
Longitudinal peak strain global	− 12.9 (3.4)	− 12.0 (3.8)	0.27	− 11.0 (3.9)	− 11.3 (5.1)	0.81	0.88 (4.06) (*n* = 27)	− 0.24 (5.32) (*n* = 29)	0.62
T2 relaxation time	50.5 (4.3)	50.0 (5.7)	0.70	48.6 (3.3)	50.2 (3.8)	0.045	− 0.51 (6.59) (*n* = 25)	1.61 (4.14) (*n* = 29)	0.30

For ECV, radial, circumferential, and longitudinal peak strain measurements, statistical significance set to *P* ≤ 0.0125 with Bonferroni correction. For T2 relaxation time, statistical significance set to *P* ≤ 0.0083 with Bonferroni correction

T2 relaxation time, a measurement of cardiac inflammation, of patients receiving placebo trended higher, suggesting worsening inflammation (48.6 ± 3.3 ms vs. 50.2 ± 3.8 ms, *P* = 0.045), between baseline and 1-year follow-up, while that of those receiving dapagliflozin did not change. However, the P-value of 0.045 was not statistically significant when compared to the corrected standard *P* = 0.0083 for multiple comparison. The representative CMRI of the same patient before intervention and at 1-year follow up are shown in Fig. 2A and ROI shown in Fig. 2B.

### Systemic inflammatory endpoints

**Fig. 3:**
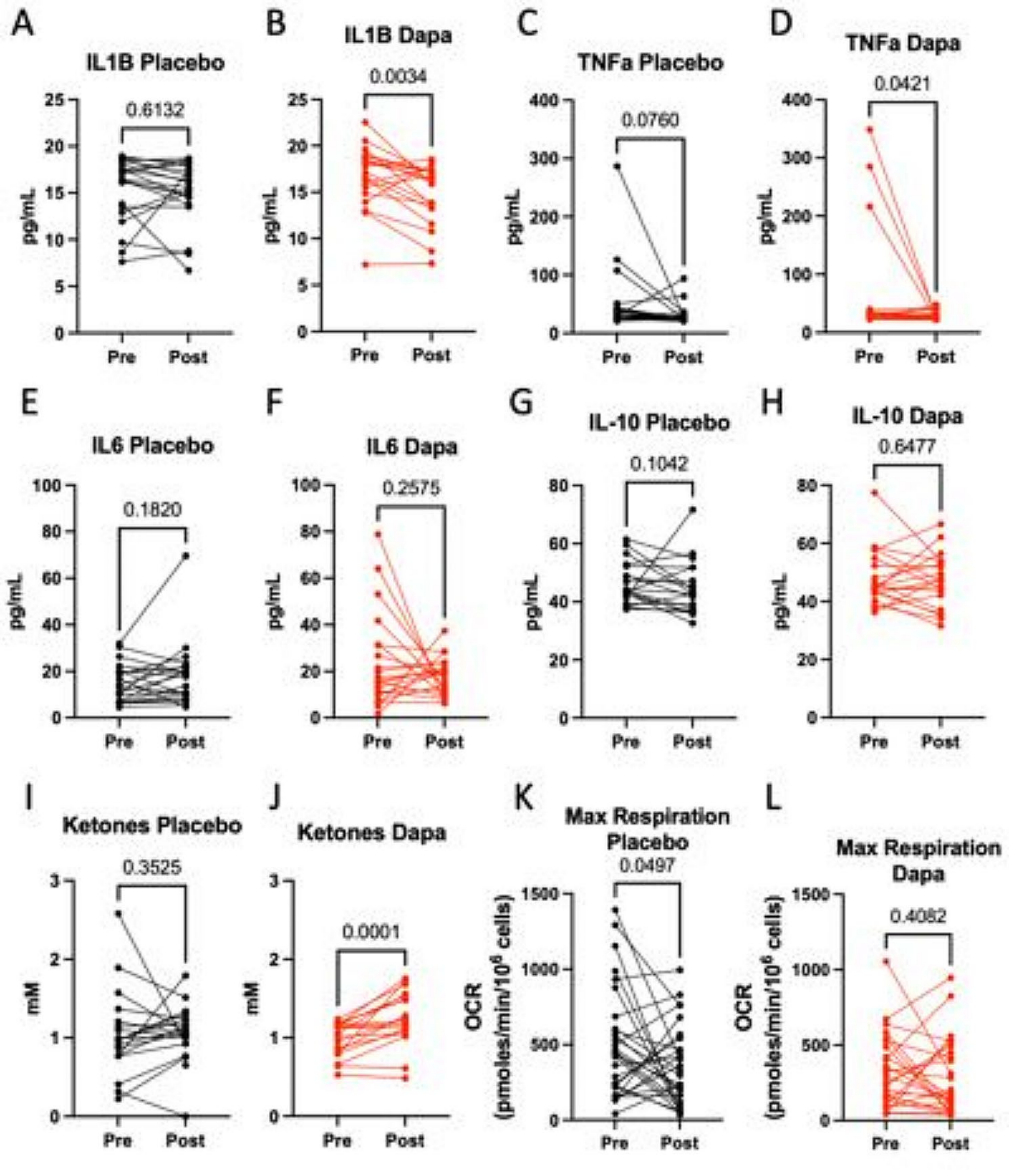
Dapagliflozin reduces systemic inflammation. Levels of pre- and post- 12-months treatment of placebo or dapagliflozin: A-H, Plasma IL-1B, TNFα, IL-6, and IL-10; I-J, Plasma ketones (acetoacetate + β hydroxybutyrate); K-L, PBMC maximal oxygen consumption rate (OCR). P-value determined by paired two-tailed T-test. Parametric t-test is used if distribution passes normality tests, otherwise non-parametric t-test is used. For plasma cytokines (IL-1B, TNFα, IL-6, and IL-10) and ketone, statistical significance set to P  ≤  0.0083 (0.05/6) with Bonferroni correction

Between baseline and 1-year follow-up, circulating CRP did not change significantly in either dapagliflozin or the placebo group (Table 3). Among the plasma proinflammatory cytokines, we observed a significant reduction in IL-1B, but not TNFα or IL-6, in patients randomized to dapagliflozin (Fig. 3A and F). IL-10, a known pro-fibrotic and anti-inflammatory cytokine, also did not change significantly in either group (Fig. 3G and H).

**Table 3:** Additional outcomes

Outcome	Drug			Placebo			Difference (1 year–baseline)		
	Baseline	1 year	P*	Baseline	1 year	P*	Drug	Placebo	P**
C-reactive protein	1.7 (1.6)	2.5 (4.1)	0.08	2.4 (0.5)	1.8 (0.3)	0.78	0.81 (2.95) (*n* = 28)	0.12 (2.20) (*n* = 29)	0.50
Glucose	159.8 (42.5)	126.8 (36.9)	0.004	164.5 (46.4)	161.0 (55.9)	0.64	− 33.04 (55.08) (*n* = 28)	− 3.48 (39.56) (*n* = 29)	0.006
A1c	7.9 (0.8)	7.4 (0.8)	0.007	7.8 (0.9)	8.0 (1.0)	0.63	− 0.52 (0.95) (*n* = 28)	0.11 (1.17) (*n* = 28)	0.012
Hematocrit	42.5 (4.0)	40.9 (3.1)	0.08	41.4 (1.8)	41.4 (2.8)	0.96	− 0.02 (0.05) (*n* = 26)	0.00 (0.02) (*n* = 29)	0.33
Body surface area	2.10 (0.21)	2.06 (0.21)	< 0.0001	2.05 (0.16)	2.04 (0.15)	0.14	− 0.04 (0.04) (*n* = 26)	− 0.01 (0.04) (*n* = 29)	0.005
BNP	37.1 (38.0)	55.1 (72.1)	0.08	31.9 (27.7)	36.4 (24.4)	0.31	17.96 (52.56) (*n* = 28)	4.58 (23.61) (*n* = 29)	0.94

All variables expressed as mean (standard deviation)

BNP Brain natriuretic peptide

*By paired t-test. **By Wilcoxon Rank Sum Test

Next, plasma ketones have been implicated as an important mediator for the beneficial effects associated with SGLT2 inhibitors, including anti-inflammation [16]. Here, we observed a significant increase of plasma ketones (β-hydroxybutyrate and acetoacetate) in patients randomized to dapagliflozin but not in the placebo group (Fig. 3I and J).

PBMC mitochondrial function is an emerging marker of sterile inflammation: a decline of PBMC maximal oxygen consumption rate (OCR), measured in the presence of mitochondrial uncoupler to facilitate maximal electron transport chain activity, is associated with increased pro-inflammatory cytokine expressions in chronic heart failure [13, 17]. Here, we observed that while the PBMC basal OCR did not change in either dapagliflozin or placebo group (Supplemental Fig. 1), the PBMC maximal OCR of patients receiving placebo declined while that of those receiving dapagliflozin did not change between baseline and 1-year follow-up (Fig. 3K and L).

Of note, although systemic inflammatory diseases were not an exclusion criterion, no patients had a known history of inflammatory diseases or was on steroidal therapy.

### Additional endpoints

In comparison to the placebo group, patients randomized to dapagliflozin demonstrated significant reductions in random serum glucose (-33 mg/dl) vs. (-3.48 mg/dl) (*P* = 0.006) and hemoglobin A1c (-0.52%) vs. (0.11%) (*P* = 0.012) between baseline and 12-month follow-up. No differences were noted in brain natriuretic peptide (BNP) (Table 3).

## Discussion

In this study, 12-months treatment of dapagliflozin reduced plasma IL-1B level, but not IL6, TNFα or serum hsCRP. To our knowledge, this is the first time that SGLT2 inhibitor is shown to lower IL-1B in a placebo-controlled double-blind randomized clinical trial in patients with T2D longer than 5 years. Furthermore, dapaglifozin significantly increased plasma ketones and attenuated the decline of PBMC maximal OCR, which was previously shown to inversely correlate with IL-1B expression in chronic heart failure [13, 17]. Despite the improvements in circulatory inflammatory endpoints, we did not observe significant changes in CMRI measures of myocardial strain, inflammation and fibrosis in patients receiving dapagliflozin. Of note, in the placebo group, there was a trend of worsening T2 relaxation time (inflammation), which was not observed in the dapagliflozin group, suggesting dapagliflozin may attenuate the progression of cardiac inflammation.

To adjust for potential confounding factors in T1 measurements [18], ECV has been shown to be a good measure of myocardial interstitial fibrosis. In the current study, we found that 12 months of SGLT2 inhibition compared to placebo did not alter ECV of T2D patients. Our findings are consistent with a prospective study which enrolled 35 T2D subjects: Before and after CMRI was performed following 6 months of Empagliflozin showed no significant effect on ECV [19]. Furthermore, in our study, SGLT2 inhibition did not change the level of plasma IL-10, a pro-fibrotic cytokine. While these results do not support the notion that the cardioprotective effects of SGLT2 inhibition is mediated via improving cardiac fibrosis, further studies are required to unravel the mechanism of action by which SGLT2 inhibitors enhance cardiac outcomes.

IL-1B is an inducible pro-inflammatory cytokine made primarily by immune cells, such as monocytes and macrophages, to function in cardiac repair as well as injury [20]. Under the NFkB-mediated transcriptional regulation, Pro-IL-1B is produced and stored intracellularly. Activation of NLRP3 inflammasome results in the cleavage and maturation of IL-1B prior to secretion. IL-1B has been shown to worsen myocardial contractile function and relaxation and induce hypertrophy [21–24]. Furthermore, in multiple animal studies, SGLT2 inhibitor is shown to antagonize the NLRP3-IL-1B axis to improve cardiovascular outcomes [4], substantiating IL-1B’s role as a potential mediator of cardiac inflammation. The recent CANTOS trial subgroup analysis provides clinical evidence that circulating IL-1B is not just a bystander but actively contributes to heart failure pathogenesis. The conjecture is corroborated by a number of small clinical trials using anakinra, a recombinant IL-1 receptor antagonist. In these studies, anakinra is shown to reduce CRP and HF hospitalization in acute STEMI patients [25, 26] and enhance exercise capacity and LVEF in HF patient with elevated CRP [27].

On the other hand, IL-6 and TNFα are known to be associated with poorly controlled diabetes and complications such as diabetic nephropathy and/or vasculopathy [28, 29]. A recent metanalysis of 18 randomized placebo-controlled clinical studies (total 5311 patients) showed that while SGLT2i lowers circulating IL6 levels, higher hemoglobin A1c levels at baseline were associated with more pronounced IL-6 reductions [30]. In contrast, the subjects of the current study had mild disease severity (baseline average Hgb A1c 7.8–7.9) without renal dysfunction (baseline average creatinine of 0.9) and only 5% with known coronary artery disease. The milder disease severity of the study subjects, together with a small sample size, likely have contributed to a lack of detectable differences in the changes of plasma IL6 and TNFα between SGLT2i and placebo groups.

PBMC mitochondrial respiration is an emerging biomarker of sterile inflammation [13, 31–33], particularly in the setting of heart failure, and was previously shown to inversely correlate with the expressions of proinflammatory cytokines, such as IL-1B [13, 17]. It is postulated that upon stimulation by circulating mitochondrial damage associated molecular patterns (MitoDAMPs), PBMC produces IL-6, leading to mitochondrial dysfunction and mitochondrial ROS production in an autocrine manner [13]. Mitochondrial ROS subsequently activates the NLRP3 inflammasome, resulting in the maturation and release of IL-1B family of cytokines [34]. In the current study, we observed that SGLT2 inhibitor sustains mitochondrial oxidative capacity of circulating immune cells, supporting the notion that dapagliflozin may reduce systemic inflammation by acting on the PBMC. As alluded, Kim et al. demonstrated that high beta-hydroxybutyrate and low insulin level have an inhibitory effect on the NLRP3 inflammasome in ex vivo macrophage experiments [6]. Together, our results are consistent with the proposed mechanism that SGLT2 inhibitor lowers systemic inflammation by increasing plasma ketones, which inhibit NLRP3 inflammasome of peripheral immune cells (PBMCs). This, in turn, prevents the maturation and release of IL-1B. Recently, transcriptomic analyses indicate that disruption of mitochondrial pathways (TCA cycle and oxidative phosphorylation) in *circulating monocytes* is a marker of elevated cardiovascular risk in T2D patients [35]. Whether and how mitochondrial dysfunction in circulating monocytes plays a role in the pathogenesis of T2D and heart failure is an area of active research.

There are several strengths of this study. First, there was excellent adherence with treatment as evidenced by significant reduction in blood glucose, hemoglobin A1c, and BMI in subjects randomized to dapagliflozin (Table 3). Second, the randomized study design was a strength, since many previous studies on SGLT2 inhibitors utilized a prospective study design. Third, the use of circulating inflammatory endpoints as well as CMRI allowed for the assessment of anti-inflammatory effect of SGLT2 inhibitor at both the systemic and organ levels.

There are several limitations and weaknesses. First, this is a relatively small clinical trial which was not powered to detect smaller effect sizes. For example, while there is a trend that dapagliflozin attenuates the progression of cardiac inflammation (T2 relaxation time), the study is underpowered to detect smaller differences. Second, previous animal studies of SGLT2 inhibition utilized direct pathologic examination of inflammation and fibrosis, whereas CMRI are known to be less sensitive in assessing these parameters. Third, the 12-month treatment period may not have been sufficient to result in detectable cardiac structural and functional changes, although in a prospective study, 3 months of SGLT2 inhibition was sufficient to demonstrate improvement in diastolic function [36]. Fourth, we did not have a matched, non-diabetic control group. Fifth, higher ECV values (greater than 32%) are associated with worse outcomes in patients with known myocarditis [37]. In contrast, the T2D cohort in our study had baseline ECV of 27–28%. To address the possibility that patients with higher baseline ECV would derive more benefits from SGLT2 inhibition, we performed a subgroup analysis in patients in the upper half of ECV values at baseline (ECV ~ 30%); however, in this cohort (*N* = 17) we still did not find a significant difference in ECV between the drug and placebo groups. Finally, the study was conducted without a sample size calculation, and hence was not powered perform subgroup analysis.

## Conclusion

In this study, we demonstrated that 12 months of daily dapagliflozin in T2D patients reduces circulating IL-1B, increases plasma ketones, and prevents the decline of PBMC mitochondrial maximal respiration, but does not improve cardiac fibrosis or strain by CMRI.

### Electronic supplementary material

Below is the link to the electronic supplementary material.

Supplementary Material 1

## Data Availability

The datasets used and/or analyzed during the current study are available from the corresponding author on reasonable request.
