# Supplementary material for: Dapagliflozin reduces systemic inflammation in patients with type 2 diabetes without known heart failure
Source: Cardiovasc Diabetol. 2024 Jun 7;23:197. doi: 10.1186/s12933-024-02294-z (PMC11161924; doi:10.1186/s12933-024-02294-z)
Supplement: Supplementary file 1 — Supplementary Material 1 [file 12933_2024_2294_MOESM1_ESM.pdf]

Supplemental Table 1. Acquisition parameters of the CMRI examination at the 3T Philips scanner.

	bSSFP cine	T1 map	T2 map	T2* map
Pulse sequence	b-FFE	TFE	GraSE	TFE
Image mode	2D	2D	2D	2D
Scan plane	Short and 3 long axis planes (two-, three-, and four-chamber)	Short axis	Short axis	Short axis
TR, ms	2.9	2.0	1RR	16.0
TE, ms	1.4	1.0	9.8	1.0
Flip angle, degree	45	20	90	25
Field of view, cm	28 x 28	30 x 30	30 x 30	30 x 30
Acquisition matrix	140 x 175	150 x 150	150 x 150	165 x 165
Resolution (interpolated) mm	0.857	1.0	1.0	1.00
Slice thickness, mm	8	10	10	10
Number of slices	12	1	1	1
Number of cardiac phases	30	1 (end-diastole)	1 (end-diastole)	1 (end-diastole)
Blood suppression	None	None	DIR	DIR
Special parameters		TI 350 ms	9 echoes	15 echoes

bSSFP – Balanced steady-state free precession

Supplemental Figure 1.

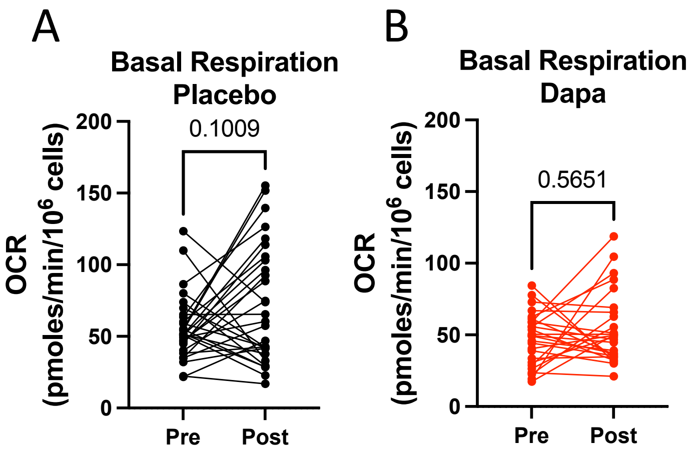

Supplemental Figure 1:

PBMC basal oxygen consumption rate (OCR). P-value determined by paired two-tailed t-test. Parametric t-test is used if distribution passes normality tests, otherwise non-parametric t-test is used.
